# In Vitro Toxicity Evaluation of Cyanotoxins Cylindrospermopsin and Microcystin-LR on Human Kidney HEK293 Cells

**DOI:** 10.3390/toxins14070429

**Published:** 2022-06-23

**Authors:** Leticia Diez-Quijada, María Puerto, Daniel Gutiérrez-Praena, Maria V. Turkina, Alexandre Campos, Vitor Vasconcelos, Ana M. Cameán, Ángeles Jos

**Affiliations:** 1 Area of Toxicology, Faculty of Pharmacy, Universidad de Sevilla, 41012 Seville, Spain; ldiezquijada@us.es (L.D.-Q.); mariapuerto@us.es (M.P.); dgpraena@us.es (D.G.-P.); camean@us.es (A.M.C.); 2 Department of Biomedical and Clinical Sciences, Faculty of Medicine and Health Sciences, Linköping University, 581 83 Linköping, Sweden; maria.turkina@liu.se; 3 CIIMAR/CIMAR—Interdisciplinary Centre of Marine and Environmental Research, University of Porto, 4450-208 Matosinhos, Portugal; amoclclix@gmail.com (A.C.); vmvascon@fc.up.pt (V.V.); 4 Biology Department, Faculty of Sciences, University of Porto, Rua do Campo Alegre, s/n, 4169-007 Porto, Portugal

**Keywords:** Cylindrospermopsin, Microcystin-LR, HEK293 cells, qRT-PCR, shotgun proteomics

## Abstract

Cyanotoxins are secondary metabolites produced by different types of cyanobacteria. Among them, Cylindrospermopsin (CYN) and Microcystins (MCs) stand out due to their wide geographical distribution and toxicity in various organs, including the kidney, which is involved in their distribution and elimination. However, the renal toxicity caused by CYN and MCs has hardly been studied. The aim of this work was to assess the cytotoxicity effects caused by CYN and MC-LR in the renal cell line HEK293, and for the first time, the influence of CYN on the gene expression of selected genes in these cells by quantitative real-time PCR (qRT-PCR). CYN caused an upregulation in the gene expression after exposure to the highest concentration (5 µg/mL) and the longest time of exposure (24 h). Moreover, shotgun proteomic analysis was used to assess the molecular responses of HEK293 cells after exposure to the individuals and combinations of CYN + MC-LR. The simultaneous exposure to both cyanotoxins caused a greater number of alterations in protein expression compared to single toxins, causing changes in the cellular, lipid and protein metabolism and in protein synthesis and transport. Further studies are needed to complete the toxicity molecular mechanisms of both CYN and MC-LR at the renal level.

## 1. Introduction

Cyanotoxins are secondary metabolites produced by different species of cyanobacteria, whose occurrence is increasing. This is due to climate change as well as eutrophication leading to their worldwide expansion [1]. Humans can be exposed to these toxins by different routes, and among them, the oral route, through the intake of contaminated water and food, is the most important. Nevertheless, parenteral, inhalation and dermal exposure may also happen [2]. 

Among cyanotoxins, the majority of studies have been conducted with Microcystins (MCs) and Cylindrospermopsin (CYN). MCs are hepatotoxins with cyclic heptapeptide structure with L-amino acid residues at the positions 2 (X) and 4 (Y), giving rise to the different variables of the molecule [3]. To our knowledge, 279 variants have been identified thus far [4], and MC-LR is the most frequently studied congener [5,6]. Their main producers are the genera *Microcystis, Planktothrix* and *Dolichospermum* [7]. The toxicity of MCs is conducted by inhibition of serine/threonine protein phosphatases, mainly PP1 and PP2A [8]. It has been also reported that MCs can be tumor promoters, and they have been classified in the group 2B by the International Agency of Research on Cancer (IARC) [9].

CYN is a tricyclic alkaloid with a hydroxymethyluracil combined with a tricyclic guanidine group [10] that can be produced by species of the genera *Chrysosporum, Dolichospermum, Raphidiopsis* and *Umezakia*, among others [11]. Its main mechanism of action is the inhibition of protein and glutathione synthesis [12,13,14].

Both cyanotoxins target the liver; however, they can also affect other organs, such as lung, thyroid, adrenal glands, intestines, nervous system [15,16,17] and also the kidney [7]. Indeed, the kidney is involved in the distribution and elimination of these toxins, and their presence in this organ was reported [7]. Regarding their toxic effects, the nephrotoxicity of MCs was recently reviewed [18]. 

In vitro, MC-LR has shown to induce, for instance, a decrease in cell viability and to cause cytoskeleton disruption and ultrastructural damage, mainly in HEK293 and Vero E-6 cell lines, from human and African green monkey origin, respectively. Moreover, Piyathilaka et al. [19] evaluated the cytotoxicity of MC-LR on two kidney cell lines (HEK293 and ACHN), and HEK293 showed the highest sensitivity. In vivo, in mice and rats, the most frequent alterations induced by MCs are changes in the activity of antioxidant enzymes as well as micro and ultrastructural damage [18]. In fish, similarly, the induction of oxidative stress and pathological changes have been also reported [20,21].

The effects of CYN on kidney, on the contrary, have been comparatively scarcely investigated. In vitro, it has been reported that CYN induces a decrease in cell viability in Vero cells [22]. In vivo studies reported that necrosis and increased lumen of proximal tubules are characteristic of renal toxicity induced by CYN in mice, as well as changes in the glomerulus [7], and changes in oxidative enzymes have been also involved as a toxicity mechanism [23,24,25]. In both cases, moreover, studies dealing with molecular mechanisms of cyanotoxins effects on the kidney are minimal or even absent.

A different point to highlight is that the toxicological evaluation of cyanotoxins, whatever the aspect investigated (hepatotoxicity, nephrotoxicity, etc.), has been mainly performed using individual compounds. However, it is known that cyanobacterial blooms are usually characterized by the presence of numerous toxins [26]. Moreover, the simultaneous occurrence in the environment of MC-LR and CYN has been reported [27,28,29]. These cyanotoxins have different structures and mechanisms of action, and phenomena, such as synergism, antagonism and/or toxicity increases must be taken into account, when their simultaneous occurrence happens. This potential interaction has been already studied in cell lines from human liver [30], nervous system [31] and genotoxicity endpoints [32,33] but not on kidney cell lines.

Therefore, considering the objective of the present work was to assess the cytotoxic effects of pure and individual CYN and MC-LR toxins in the human kidney cell line HEK293. Moreover, in order to add some insights into CYN mode of action, we evaluated the alterations in the expression of selected genes involved in metabolism and mechanisms of toxicity by real-time quantitative PCR (qRT-PCR) of this toxin for the first time in these renal cells. Finally, it is innovative to investigate the individual and combined effects of CYN and MC-LR in HEK293 cells by post-genomics analysis using shotgun proteomics.

## 2. Results

### 2.1. Viability of HEK293 Cells Exposed to Cyanotoxins

**Figure 1:**
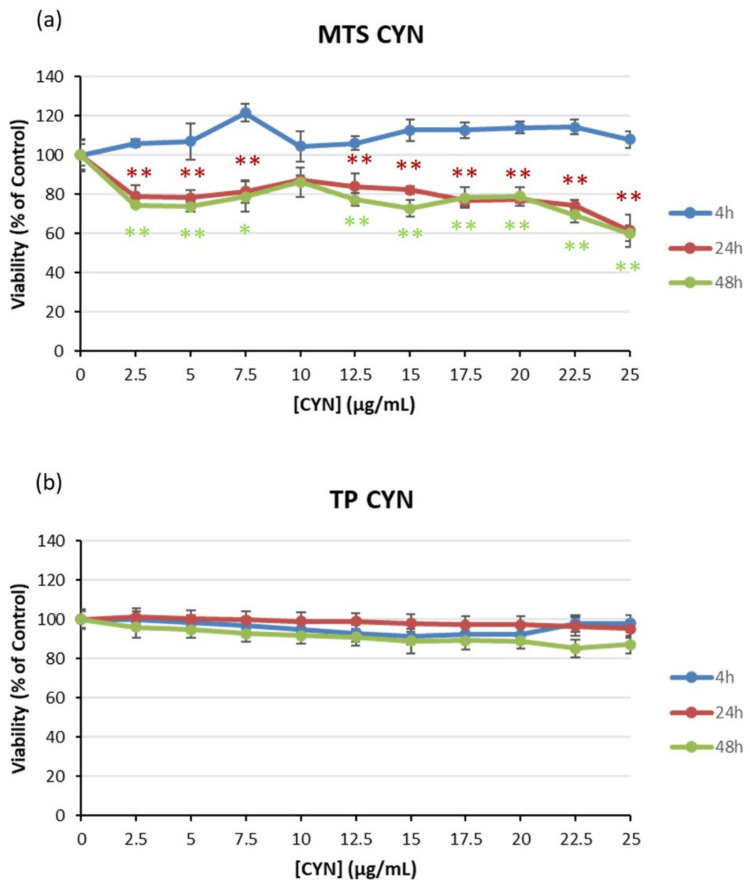
The effect of CYN on the viability of HEK293 cells. Viability was determined with the MTS assay (**a**) and TP (**b**) after exposure to 0–25 µg/mL CYN for 4, 24 and 48 h. Significantly different from the control group * *p* < 0.05 and ** *p* < 0.01.

When HEK293 cells were exposed to CYN (0–25 µg/mL), no effects were observed in the MTS reduction assay after 4 h. At 24 and 48 h, however, the viability slightly decreased at all concentrations assayed (Figure 1a). The highest concentration resulted in a viability of 60%. On the contrary, the total protein (TP) content of the cultures was not altered at any of the exposure times and concentrations assayed (Figure 1b). Therefore, no EC_50_ values could be derived from any of the endpoints considered.

**Figure 2:**
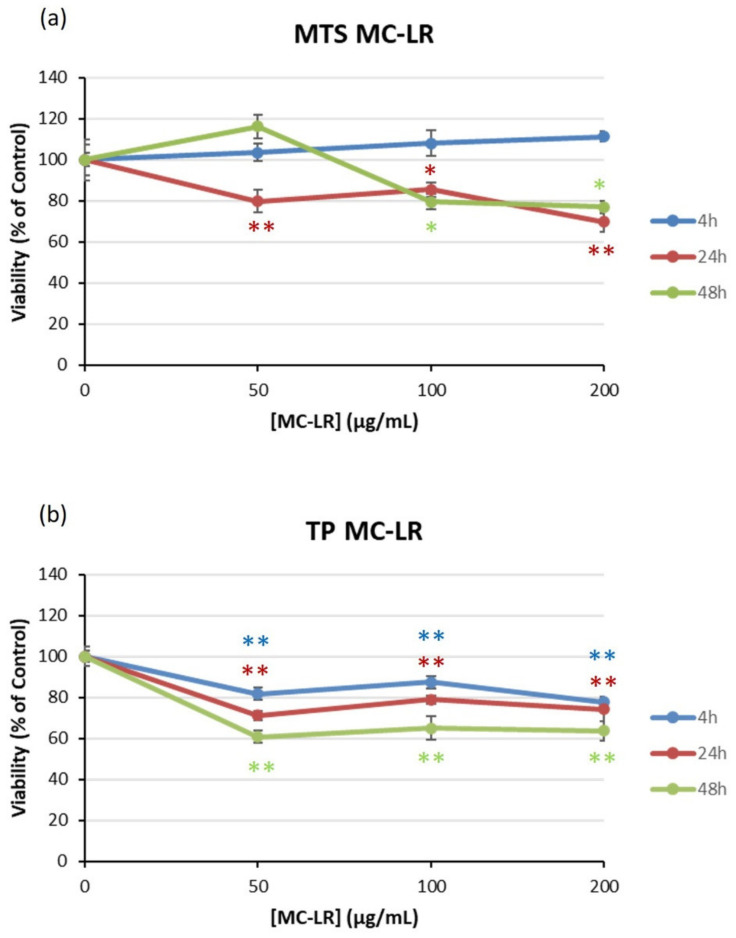
The effect of MC-LR on the viability of HEK293 cells. Viability was determined with the MTS assay (**a**) and TP (**b**) after exposure to 0–200 µg/mL MC-LR for 4, 24 and 48 h. Significantly different from control group * *p* < 0.05 and ** *p* < 0.01.

With respect to MC-LR, cells were exposed to 0–200 µg/mL for 4, 24 and 48 h of exposure. In this case, cell viability decreased to 80% after 24 and 48 h in the MTS assay (Figure 2a) and to 60% in the protein content assay after 48 h (Figure 2b). Again, no EC_50_ values could be derived.

### 2.2. Effect of CYN on mRNA Expression

The mRNA expression of selected genes was assessed after exposure to 0.5 and 5 µg/mL CYN for 4 and 24 h by quantitative real-time PCR (Table 1). In general terms, the values showed mainly upregulation in the expression of genes after the longer exposure time (24 h) and the highest concentration (5 µg/mL). This is the case for genes involved in xenobiotic metabolism (*CYP1A1* and *CYP1A2*), DNA damage response (*TP53* and *CDKN1A*), oxidative stress (*CAT* and *GPX1*) and apoptosis/survival (*BCL2*). A lower number of genes showed an altered expression pattern after 4 h: upregulation in *CYP1A1* and *SOD1* and downregulation in *CAT*, all of them again at the highest concentration evaluated. The lower concentration (0.5 µg/mL) changed the expression of *CYP1A2*, *CDKN1A* and *GPX1* after 24 h. The only gene that was not altered by any treatment was *BAX*.

### 2.3. Proteomics Analysis

**Figure 3:**
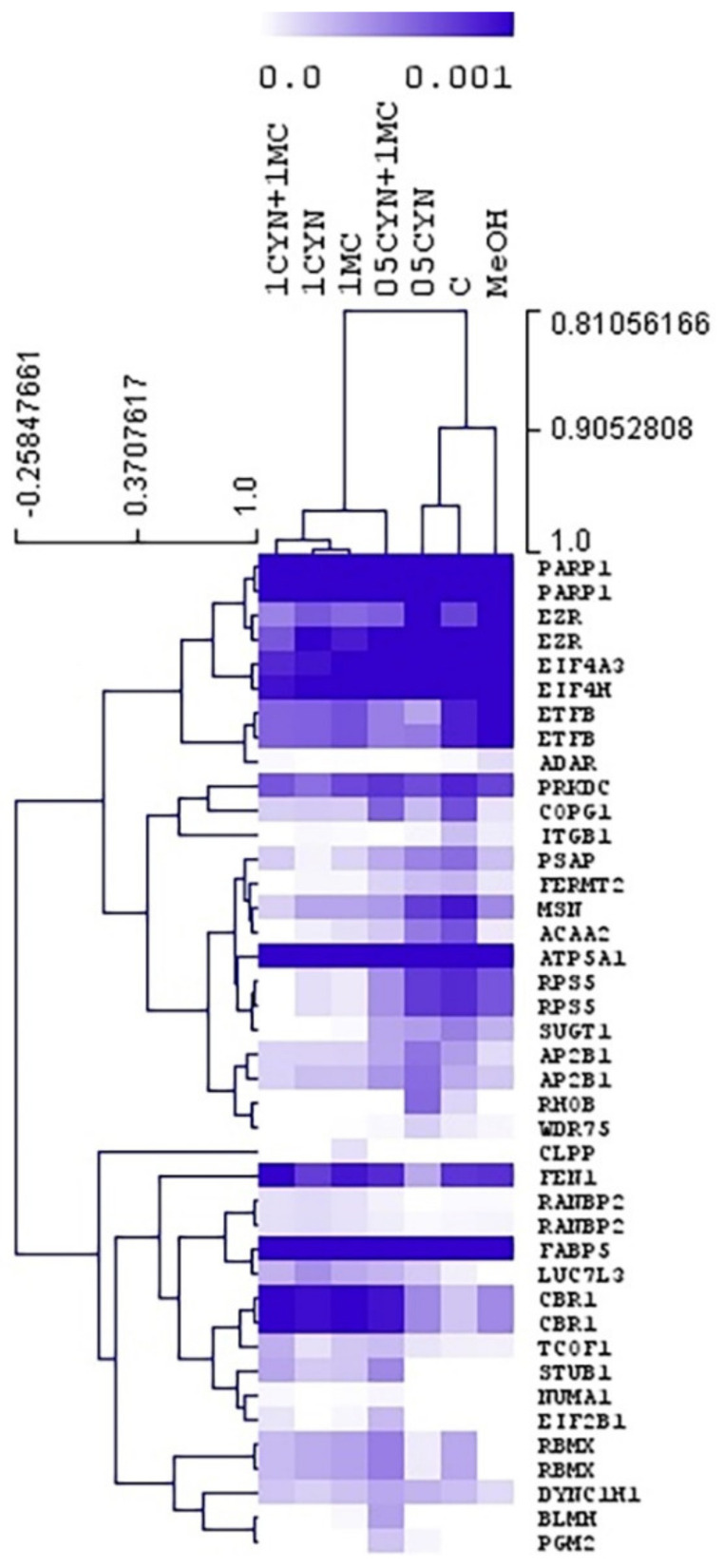
Analysis of HEK293 cells after exposure to cyanotoxins. The color map symbolizes the relative expression of proteins displaying quantitative variations among experimental groups (ANOVA, *p* < 0.01). Proteins are presented in lines and group samples in columns. Control group (C), MeOH (solvent control group), group exposed to Cylindrospermopsin (CYN), group exposed to Microcystin-LR (MC) and group exposed to the combination of Cylindrospermopsin and Microcystin-LR (CYN + MC). Numbers preceding group abbreviations indicate toxin concentrations.

To assess the molecular responses of HEK293 cells exposed to cyanotoxins, a quantitative shotgun proteomics analysis was performed. Forty one proteins displayed quantitative differences among experimental groups (ANOVA, *p* < 0.01), and 17 proteins showed statistical differences in comparison to the control group. The overall differences in protein expression are reported in the heatmap in Figure 3. Some proteins were presented in more than one experimental group. The sample group cluster analysis in Figure 3 revealed that the experimental group that displayed more variations with respect to control was the one exposed to the highest concentrations of both cyanotoxins (1CYN + 1MC). Moreover, control and MeOH groups are positioned closer to each other, thus, indicating that the effect of MeOH in the experiment was minimal. The relative expression levels of proteins were also analyzed using hierarchical clustering (Figure 3).

Table 2 shows the 17 proteins displaying differences with respect to the control group. This group of proteins will be most relevant to describe the effects of exposure to cyanotoxins. Proteins modified after cyanotoxin exposure include proteins related to RNA-binding and mRNA and protein transport (E3 SUMO-protein ligase RanBP2-RANBP2), Golgi structural integrity and intracellular protein transport (Coatomer subunit gamma-1-COPG1), immunity and host–virus interaction (Moesin-MSN), regulation of lipid metabolic process and enzyme activator activity (Prosaposin-PSAP), mRNA binding and mRNA splice site selection (Cisplatin resistance-associated overexpressed protein and isoform CRA_b-LUC7L3), lipid metabolism (3-ketoacyl-CoA thiolase and mitocondrial-ACAA2), ubiquitination and proteasomal degradation of target proteins (Protein SGT1 homolog-SUGT1), host cell receptor for virus entry and cell adhesion (Integrin beta-1 and ITGB1), hydrolase function and proteolysis involved in cellular protein catabolic process (ATP-dependent Clp protease proteolytic subunit and mitochondrial-CLPP), proteins with functions connected with gene transcription/translation and RNA-binding protein (40S ribosomal protein S5-RPS5), catalytic activity (carbonyl reductase [NADPH] 1-CBR1), regulation of RNA polymerase I (Treacle protein, TCOF1), metabolic inactivation of the antitumor drug Bleomycin (Bleomycin hydrolase-BLMH), cell adhesion (Fermitin family homolog 2-FERMT2), proteasomal degradation and protein modification (E3 ubiquitin-protein ligase CHIP-STUB1), initiation factor and protein biosynthesis (translation initiation factor eIF-2B subunit Alpha-EIF2B1) and catalytic activity (phosphoglucomutase-2, PGM2).

Simultaneous exposure to both cyanotoxins led to alterations in the expression of a higher number of proteins in comparison to the other experimental conditions. These included proteins involved in processes such as Golgi structural integrity and intracellular protein transport (*COPG1*), immunity, host–virus interaction and host cell receptor for virus entry as cell adhesion (*MSN*, *ITGB1* and *FERMT2*). Furthermore, other proteins affected were proteins associated with lipid metabolism (*PSAP* and *ACAA2*), ubiquitination, proteasomal degradation and protein modification (*SUGT1* and *STUB1*). Proteins that participated in catalytic activity (*CBR1* and *PGM2*), gene transcription/translation and RNA-binding protein (*RPS5*), regulation of RNA polymerase I (*TCOF1*), metabolic inactivation of Bleomycin (*BLMH*) and protein biosynthesis and initiation factor (*EIF2B1*) were also altered.

The results obtained showed that the protein expression profile of HEK293 cells was affected after cyanotoxins exposure. Therefore, cyanotoxins can modify the proteome of this renal cell line.

## 3. Discussion

Previous in vivo experiments indicated that the kidney was the most affected organ in several experimental models, such as mice [34], fish exposed to lyophilized *A. ovalisporum* cells containing CYN at relevant concentrations (10–100 µg/L) [25] or pure CYN [23]. However, regarding in vitro studies performed in renal cells with cyanotoxins, dealing with the potential toxicity mechanisms of these toxins is rare. In the specific case of HEK293 cells, up to now, the studies have been performed with MCs, in particular MC-LR [19,35,36,37].

The present study provides evidence that CYN and MC-LR are able to induce a cell viability decrease in HEK293 cells, with CYN being more cytotoxic in comparison to MC-LR. For both cyanotoxins, no EC_50_ were derived from the MTS assay, and in the case of MC-LR (1–200 µM), the cell viability decreased to 80% after 24 and 48 h, with a small increase at the lowest concentration, potentially due to a hormetic effect [38]. Hormetic effects by MC-LR have been reported for MC-LR in kidney cell lines HEK293 [39] and Vero-E6 [40]. These results for MC-LR showed less sensitivity than those previously obtained in the same cell line (HEK293 cells) and in the human kidney adenocarcinoma (ACHN) cell line by Piyathilaka et al. [19]. 

These authors used the same concentrations of MC-LR (1–200 µM) and determined the cytotoxicity by 3-(4,5-dimethylthiazol-2-yl)-2,5-diphenyltetrazolium bromide (MTT) and sulphorhodamine B (SRB) assays. In both cases, cell viability was significantly decreased at 50 µM for 24 h. The higher cytotoxicity reported for MC-LR in comparison to the present work could be due to the culture conditions, cytotoxicity assays applied, etc. In other renal cells, monkey kidney cell line (Vero E6), a marked cytotoxicity of MC-LR was reported from 25 µM for 24 h by the MTT assay [40]. 

Li et al. [39] reported that MC-LR at low concentrations stimulated PP2A, whereas at high concentrations, induced an inhibition in HEK293 cells. The effects on PP2A/C led to destabilization of cytoskeleton, cell detachment and possibly further anoikis, a form of apoptosis induced by cell detachment from the extracellular matrix. Moreover, experiments with OATP-transfected HEK293 cells reported EC_50_ values for MC-LR of 214–257 nM, with lower EC_50_ values of the more hydrophobic MC congeners [35]. In general, regarding MC-LR exposure, the low cytotoxicity observed could be partially attributed to the absence of specific OATP transporters. However, all the reports above mentioned demonstrated that MC-LR is able to induce toxicity in HEK293 cells.

Regarding to CYN, there are no studies available with HEK293 cells; however, Froscio et al. [22] evaluated its cytotoxicity in different cells lines, including Vero (from African green monkey kidney). They observed that CYN induced a delayed toxicity in Vero cells and that this cell line was less sensitive than hepatic cell lines but more sensitive than the intestinal cell line Caco-2. 

Moreover, Froscio et al. [41] reported that CYN uptake in Vero cells was slow but sufficient to induce cytotoxicity. More recently, Moraes et al. [42] observed that CYN induced necrosis in all concentrations assayed (up to 1 µg/mL) in LLC-PK1 kidney tubular cells from pigs. This work reported for the first time in HEK293 cells at the molecular level that non-cytotoxic concentrations of CYN can modify the regulation of different genes. Similarly, the changes in the protein expression profile of HEK293 cells after exposure to individual MC-LR or CYN, as well as to both cyanotoxins simultaneously, are reported in a novel way in order to clarify the mechanisms involved in cyanotoxin renal toxicity.

Studies about the modulation of gene expression after CYN exposure are limited. Several works have evaluated the influence of CYN on the alteration of mRNA levels of genes involved mainly in the CYN metabolism, response to DNA damage and repair, oxidative stress, survival and/or cell death in several in vitro models, such as human peripheral blood lymphocytes (HPBLs) [43] and HepG2 cells [44,45], including the advanced 3D cell model developed from these cells [46]. In addition, other works with CYN have been conducted in vascular smooth muscle cells (VMSCs) [47] and in human umbilical vein endothelial cells (HUVECs) [48]. Changes in the expression of these genes were also induced in hepatic cells, HepG2, by binary mixtures of MC-LR/CYN [32,49], or CYN and Bisphenols [46]; however, to date, no previous studies have been performed in HEK293 cells.

In the present study, upregulations of *CYP1A1* and *CYP1A2* at the highest CYN concentration (5 µg/mL) assayed, mainly after 24 h of exposure, were reported, which were approximately 24-fold and 11-fold increased, respectively. These results are in agreement with those previously found in HPBLs and HepG2 cells [43,44], while the transcription of *CYP1A2* was not significantly affected in the HepG2 3D cell model, with *CYP3A4* upregulated [46], and this confirms the evidence that CYP-450 family enzymes are involved in the biotransformation of CYN [44]. 

*CYP1A1* is one of the main cytochrome P450 enzymes, extensively studied for its capacity to activate compounds with carcinogenic properties [50]. *CYP1A2* is also involved in the metabolism of several drugs and endogenous compounds. It is of particular interest because of its crucial role in chemical carcinogenesis and its susceptibility to induction at transcriptional and translational levels [51]. Our results provide the first evidence that exposure to CYN induces transcription of these genes in HEK293 cells and confirm previous research that they are involved in CYN metabolic activation to genotoxic and potential carcinogenic effects [44].

In addition, we found upregulation in the expression of the P53 tumor-suppressor gene after 24 h CYN exposure at the highest concentration assayed in HEK293 cells. The tumor-suppressor gene, p53, plays a central role in the cellular response to xenobiotics in general, which damages DNA by activation of transcription of several essential genes controlling cell cycle arrest/DNA repair, senescence, differentiation and apoptosis [52]. These changes in p53 gene levels are in agreement with results reported previously in HPBLs after 24 h of exposure to CYN [43]. 

In contrast, in HepG2 cells, CYN did not induce any change in the expression of P53, at any assayed concentrations after 4–24 h of exposure [44], and Bain et al. [53] only detected P53 protein accumulation after 48 h of exposure. Moreover, the main target of P53 upon DNA damage is *CDKN1A* at node p21, an inhibitor of cyclin-dependent kinases (CDKs) that inhibits the cell cycle at the G1/S and the G2/M transitions [52,54]. In the present study, CYN upregulated the expression of *CDKN1A* gen only after 24 h of exposure (no changes at 4 h CYN-exposure), which correlates well with previous reports in other experimental cell lines [43,44,45,53]. 

Furthermore, Zegura et al. [43] showed that after 24 h of exposure of HPBLs to CYN, some p53-downstread regulated genes, such as *MDM2* and *GADD45α,* were upregulated, whereas no differences in the *CDKN1A* mRNA levels were found in comparison to control cells. 

In this work, the gene expressions were measured at only two time-points (4 and 24 h), similarly to the present study, and the authors indicated a later induction of *CDKN1A* in comparison to other genes. The upregulation of *CDKN1A* was also reported in HepG2 spheroids, indicating DNA damage effects of CYN [46]. Oxidative stress is one of the recognized CYN toxicity mechanisms, as it has been demonstrated in several in vitro studies available in the scientific literature [55] and in vivo models [23,24] at environmentally relevant concentrations [25]. 

Among the enzymes involved in oxidative stress, the superoxide dismutase-catalase (SOD-CAT) system provides the first defense against oxygen activity [56], because SOD catalyzes the dismutation of the superoxide radical to molecular oxygen and hydrogen peroxide, which is detoxified by the *CAT* activity, and a simultaneous induction response in the activities of both enzymes has been observed in fish exposed to CYN in vivo [25]. 

In this experiment from our lab, the initial increase of SOD activity detected in the kidney of fish was followed by a decrease after 14 d of exposure, indicating an increased susceptibility of the kidney in comparison to the liver. Moreover, *CAT* activity appeared to be more active trying to control the oxyradicals generated [25]. However, studies on the gene expression profile of these antioxidant enzymes are scarce. Up to now, only Zegura et al. [43] demonstrated significant upregulation of *SOD1*, while *CAT* was not changed after 24 h exposure of HPBLs to CYN. 

Recently, Zhang et al. [47] reported the promotion of the expression of *SOD1, CAT* and *GPX1* in vascular smooth muscle cells (VMSCs) at 24 h of exposure (200–2000 nM CYN). In the present work, *SOD1* was upregulated after 4 h of exposure, whereas *CAT* showed significant changes after 4 and 24 h of exposure, mainly upregulation after 24 h (three-fold in comparison to the controls). These results confirm that CYN is able to induce oxidative stress in these renal cells. Several studies reported that *SOD, CAT* and *GPX* are important antioxidant enzymes for the maintenance balance [25,57]. *CAT* is globally considered the key antioxidant enzyme that decomposes H_2_O_2_ to O_2_ and H_2_O [58], thus showing, in the present work, a higher sensitivity of its gene expression in comparison to *SOD1*. *SODs* are involved in dismutation of the highly superoxide anion to O_2_ and to the less reactive species H_2_O_2_ [59]. 

In addition to *CAT*, the glutathione peroxidase family (*GPX*) of enzymes acts cooperatively as scavengers of hydrogen peroxide (both enzymes) and other hydroperoxides (*GPX*). In general, *GPX* reduces hydrogen peroxide as well as organic hydroperoxides to water and/or the corresponding alcohol [60]. In the present study, the gene expression of the enzyme *GPX1* was significantly upregulated after 24 h exposure of cells to CYN, confirming the oxidative stress induction by this toxin. 

This result is in agreement with the upregulation in the expression of genes *GPX1* after 24 h of exposure in HPBLs, while the mRNA level of *CAT* was unchanged [43]. In addition, in vivo, the gene expression of *GPX* in the kidney of tilapia exposed to CYN was also increased significantly after exposure to CYN (200–400 µg/kg CYN), thus, confirming the induction of oxidative stress by CYN in kidney [23]. Globally, in HEK293 cells, the upregulation of *CAT* and *GPX1* at 24 h and *SOD1* after 4 h of CYN exposure in the present work contributes to understanding and confirming the role of the oxidative stress as the mechanism responsible for CYN pathogenicity in these renal cells.

In relation to the genes involved in apoptosis, the p53 protein is a major regulatory of cellular response to various types of stress, and P53-mediated apoptosis is related to the regulation of the transcription of the *BCL*-family [48]. The *BCL*-2 family includes a network of pro-and anti-apoptotic proteins, and among them, *BAX*, a pro-apoptotic protein (cytosol) could be activated to induce cell death. Moreover, *BCL*-2, an anti-apoptotic protein, mainly localized in the mitochondrial membrane, could interact with *BAX* to form heterodimers to prevent mitochondrial changes during apoptosis [48]. 

In this study, the gene expression profile of *BAX* was not significantly affected in HEK293 cells after CYN exposure, whereas the toxin induced the expression of *BCL*-2 gene at the highest concentration assayed (5 µg/mL) after 24 h (3-fold), thus, leading to a lower ratio of *BAX*/*BCL*-2 at these conditions. Consequently, the apoptosis would not be affected. Regarding the scientific literature, contradictory results have been found in the deregulation of pro- and anti-apoptotic genes after CYN exposure. Thus, up-regulation of both genes after 24 h of exposure of HPBLs to CYN has been indicated, being more pronounced for *BCL*-2, with a ratio of *BAX/BCL*-2 in favor of *BCL*-2, thereby, indicating the suppression of apoptosis at the same assayed concentration of the present work (0.5 µg/mL) [43]. These authors also reported in HepG2 cells that CYN exposure significantly deregulated five of the six selected genes encoding proteins from the *BCL*-2 family; specifically, the toxin down-regulated the anti-apoptotic *BCL-2*, while *BAX* was upregulated by CYN for less than 1.5 fold [45]. 

Recently, CYN promoted the expression of *BAX* and *BCL*-2 genes in HUVEC cells, with differences in the ratio of both genes depending on the CYN concentrations: significant increases in the 20 and 200 nM CYN-treated groups and no significant differences in the 2 and 2000 nM CYN-exposed groups [48]. In VMSC cells, Zhang et al. [47] reported the promotion of cellular apoptosis by CYN with induction of the expression of p53 and *BAX* genes and inhibition of the expression of *BCL*-2 gene, leading to a higher ratio of *BAX*/*BCL*-2. Finally, in the HepG2 3D cell model, Hercog et al. [46] reported that CYN significantly deregulated genes involved in cell proliferation and apoptosis (BBC3) after 72 h exposure (0.5 µg/mL), suggesting that one of the mechanisms of CYN action is the induction of apoptosis.

Globally, the discrepancies found in the studies reported could be due to the experimental model employed, the concentrations of CYN assayed or the time of exposure. In any case, the results demonstrate that CYN induced effects in kidney cells at the molecular level. As far as we know, the molecular process that describes the response of HEK293 cells after exposure to cyanotoxins, as it is the case of CYN and MC-LR, has been scarcely studied thus far. This work investigates for the first time the effects caused in the protein expression of HEK293 cells after exposure to CYN, MC-LR and their combination. 

The assessment of proteins, genes and other biomolecules through the use of OMICS techniques, such as proteomics, allows the study to the molecular pathways and metabolism of organisms in a more comprehensive manner [61]. In environmental sciences, proteomics enables to investigate and disclose the toxicity of many xenobiotics or pollutants in the biota [62]. In addition, the existence of new methods of sample preparation, such as filter-aided sample preparation (FASP), is making proteomics studies more trustworthy and sensitive [61].

In our study, proteomics results indicated that HEK293 cells that were simultaneously exposed to both cyanotoxins (CYN + MC) showed more differences at the proteome level in comparison to cells exposed to individual toxins (CYN or MC-LR) (Figure 3 and Table 2). This response can be due to a synergetic response of CYN and MC-LR in the metabolism of cells. These results are not in line to those previously described by Gutiérrez-Praena et al. [30], which showed an antagonistic response when the cytotoxicity of both pure cyanotoxins in combination was assessed in HepG2 cells or in SH-SY5Y cell line [31]. 

Moreover, it has been proven that the genotoxic potential observed in HepG2 cells after the simultaneous exposure to CYN and MC-LR is comparable to that of CYN alone [32,33,49]. This fact is in agreement with the results obtained in the present study, since, although significant differences in protein expression were observed mainly in the groups exposed to the combination CYN + MC, protein expression differences were also reported in cells exposed to CYN and MC-LR alone (Figure 3 and Table 2), with CYN affecting more proteins than MC-LR, suggesting that cells are more sensitive to this toxin.

Globally, the proteomics results in the present work indicate a different response of HEK293 cells depending on the exposure group (Figure 3 and Table 2). Cells exposed to CYN + MC affected mainly mechanisms associated with lipid metabolism and proteins with functions related with cytoskeleton structure, chaperone, cell adhesion and protein translation. 

The diverse protein expression in HEK293 cells exposed to the highest concentrations of both cyanotoxins (Figure 3 and Table 2) could be interpreted as the interaction between CYN and MC-LR with the proteins, which prompt different molecular responses. In the case of *CBR1*, an increase in the expression of this protein was observed after exposure to the combination of both cyanotoxins in comparison to control group. This protein is a NADPH-dependent carbonyl reductase with broad substrate specificity and catalyzes the reduction of an extensive diversity of carbonyl compounds, including quinones and prostaglandins, among other compounds [63]. 

Other proteins affected were *PSAP* and *ACAA2*, which are involved, respectively, in the following processes: lysosomal degradation of sphingolipids and mitochondrial beta-oxidation pathway. Thus, these proteins play an important role in the lipid metabolism. Furthermore, the expression of different proteins, such as *MSN*, *SUGT1*, *ITGB1*, *RPS5* and *TCOF1*, were also altered showing a decrease in most of them after exposure to both cyanotoxins. These proteins are involved in the following functions: immunity and host–virus interaction, protein ubiquitination and proteasomal degradation of target proteins, host cell receptor for virus entry and cell adhesion, functions linked with gene transcription/translation and RNA-binding protein and the regulation of RNA polymerase I. 

Therefore, these proteins play a critical role in the regulation of many other proteins in the cell. In addition, *COPG1* was also altered after the simultaneous exposure to CYN + MC-LR showing a decrease in its expression. This protein interacts with a cytosolic protein complex, which is involved in the Golgi structural integrity and in intracellular protein transport. Moreover, is also implicated in the cell processing, activity and endocytic recycling of low-density lipoprotein (LDL) receptors, being required for controlling lipid storage in lipid droplets. Another protein whose expression was affected is *FERMT2*, a protein with putative functions in cell adhesion, cell surface signaling and regulation of cell differentiation and cellular component biogenesis. In the present work, a significant decreased was observed after exposure to the highest concentration of CYN + MC.

When cells were exposed only to CYN, several of the above-mentioned proteins were also affected, namely *PSAP*, *ACAA2*, *MSN*, *SUGT1*, *ITGB1* and *COPG1*. Moreover, the results showed that CYN alone induces changes in the expression of proteins involved in mRNA binding and mRNA splice site selection (*LUC7L3*) and RNA-binding and mRNA and protein transport (*RANBP2*) (Figure 3 and Table 2). Previous in vivo studies conducted with CYN + *C. raciborkii* cells showed that CYN affected different biochemical pathways related to energy production, mitochondrial function and metal transport [64].

In the case of the group exposed to MC-LR alone, few differences with respect to the control group were observed, with only three proteins altered: *ITGB1*, *COPG1* and *CLPP* (Table 2). In the last case, the expression of *CLPP* was only changed after exposure to MC-LR and not by CYN or CYN + MC-LR. This protein is involved in hydrolase function, and proteolysis implicated in cellular protein catabolic process. These results demonstrate that MC-LR is able to induce a response in HEK293 cells despite the lower sensitivity attributed to this cell line due to its lack of specific OATPs. Pure MC-LR standard was used, and thus the effects observed can be attributed to the toxin. 

Menezes et al. [65] also observed that MC-LR induced toxic effects in the kidney Vero-E6 cell line, for which no previous studies had reported the expression of OATPs, or that an alternative transport system was involved in the microcystin uptake by kidney cells. They explained that the type and extension of these effects were highly dependent on the toxin concentration and that, for example, certain responses, such as necrosis or apoptosis, required much higher concentrations than others (i.e., mitogen-activated protein kinase (MAPK) expression). Similarly, in our study, cell viability was a less sensitive biomarker than proteomic analysis. In any case, the potential mechanisms of toxicity of MC-LR on cell lines where the presence of OATPs is not confirmed remains an open issue and should be further investigated.

Finally, the STRING analysis established a functional link between *MSN*, *ITGB1* and *FERMT2* (Supplementary Figure S1). This protein cluster may well reflect one of the main molecular effects of CYN and CYN + MC-LR in HEK293 cells. These proteins share functions in cell adhesion (integrin-mediated cell adhesion) and cytoskeletal functions. Furthermore, alterations on these proteins and processes could have major implications in the functions of cells, tissues and organs.

Here, we report the molecular effects associated with a subtoxic exposure to cyanotoxins MC-LR and CYN. In fact, little is known of the subtoxic effects of cyanotoxins at the molecular level and especially on the protein expression. To date, proteomic research has been reporting molecular responses associated with the toxic effects caused by MCs and CYN toxins of increased adversity and associated to apoptosis, cytotoxicity or genotoxicity. These works revealed molecular responses triggered by MCs involving pathways related with signal transduction, apoptosis, protein degradation, cell cycle, cell differentiation, transporter, oxidative stress and energy metabolism [66,67,68,69] in cell and animal models. 

These studies enabled the identification of the p53 protein as a potential target of MCs [66] and BID-*BAX*-*BCL*-2 as the main mechanism of apoptosis mediated by this cyanotoxin [70]. Proteomic alterations associated with exposure to CYN are less known. Liebel et al. [71] reported alterations in proteins involved in different biological processes: protein folding, xenobiotic efflux, antioxidant defense, energy metabolism and cell anabolism, cell signaling, tumorigenic potential and cytoskeleton structure. 

The proteins in HepG2 cells affected by CYN included G protein-coupled receptors (GPCRs), heterogeneous nuclear ribonucleoproteins (hnRNP), MRP3 and glutathione peroxidase, which can be associated with cell protection against some chemicals and ROS [71]. Our work expands our understanding of CYN toxicity by establishing the proteins *MSN*, *ITGB1* and *FERMT2* as possible targets, as these play relevant roles in cell adhesion (integrin-mediated cell adhesion) and cytoskeleton functions.

## 4. Conclusions

This work shows for the first time the nephrotoxic effects caused by CYN and MC-LR individually and simultaneously in the HEK293 renal cell line. Alterations in gene expression (*CYP1A1, CYP1A2, TP53, CDKN1A, GPX1* and *BCL2*) were observed mainly at the highest concentration assessed (5 µg/mL) and after 24 h of exposure. Moreover, shotgun proteomics was used to assess alterations in the protein expression profile, and the greatest changes were observed after simultaneous exposure to CYN and MC-LR. 

Among the proteins affected, the corresponding genes are involved in cellular metabolism (*CBR1* and *PGM2*), lipid metabolism (*PSAP* and *ACAA2*), cell adhesion (*ITGB1* and *FERMT2*) and different functions at the protein level, such as *MSN*, *BLMH*, *STUB1*, *SUGT1*, *EIF2B1*, *TCOF1*, *RPS5* and *COPG1*, were implicated in protein metabolism, regulation synthesis and transport. These results allow a better understanding of the effects of these cyanotoxins in renal cells at the molecular level, particularly regarding protein expression. Furthermore, additional studies are needed to investigate the toxicity caused by these cyanotoxins at the renal level and to clarify the molecular mechanisms involved.

## 5. Materials and Methods

### 5.1. Supplies and Chemicals

Microcystin-LR standard (99% purity) and Cylindrospermopsin standard (95% purity) were obtained from Enzo Life Sciences (Farmingdale, New York, NY, USA). Culture medium, foetal bovine serum (FBS) and cell culture reagents were obtained from Biomol (Sevilla, Spain). MTS assay (CellTiter 96 AQueous One Solution Cell Proliferation Assay) was supplied by Promega Biotech Iberica S.L. (Madrid, Spain). TRIzol^®^ reagent was from Gibco BRL (Paisley, Scotland) and benzo(a)pyrene (B(a)P) was obtained from Sigma-Aldrich (St. Louis, MO, USA). 

High-capacity cDNA Archive Kit and Taqman Gene Expression Assays were from Applied Biosystems (Forest City, CA, USA), TaqMan Universal PCR Master Mix from Applied Biosystems (Branchburg, NJ, USA) and Human GAPDH from Applied Biosystems (Forest City, CA, USA). Tris(hydroxymethyl)aminomethane (Tris), Sodium Dodecyl Sulfate (SDS), Dithiothreitol (DTT) and protease inhibitors (Halt PI Cocktail CAT #78429,Thermo Scientific, Waltham, MA, USA). Centrifugal filter units with nominal molecular weight limit (NMWL) of 30 kDa (MRCF0R030) were from Millipore, Billerica, MA, USA. Iodoacetamide (IAA), Trypsin (CAT #3708985001, Roche, Mannheim, Germany), Reversed phase extraction tips (C18 Tips 100 µL) were from Thermo scientific. The rest of the chemicals were purchased in Sigma-Aldrich (Madrid, Spain) and VWR International Eurolab (Barcelona, Spain).

### 5.2. Cell Culture and Treatment

HEK293 cells, derived from human embryonic kidney, were obtained from the American Type Culture Collection (CRL-1573). These cells were maintained in DMEM medium supplemented with 10% FBS, 1% L-glutamine 200 mM, 1% sodium pyruvate, 1% non-essential amino acids and 1% penicillin/streptomycin solution, in an atmosphere containing 5% CO_2_ at 95% relative humidity at 37 °C (CO_2_ incubator, NuAire^®^, Plymouth, MN, USA). Cells were grown 80% confluent in 75 cm^2^ plastic flasks and harvested twice a week with 0.25% trypsin-EDTA (1×).

### 5.3. Cytotoxicity Assays

For the cytotoxicity assays, HEK293 cells were seeded at a density of 5 × 10^5^ cells/mL in 96-well culture plates and incubated at 37 °C and 5% CO_2_ for 24 h before the exposure was performed. From the stock solution of 4000 µg/mL MC-LR, serial dilutions were prepared from 0 to 200 µg/mL MC-LR. In the case of CYN, from the stock solution of 1000 µg/mL CYN, serial dilutions were prepared from 0 to 25 µg/mL CYN. The concentrations were selected in order to obtain mean effective concentration (EC_50_) values according to Pichardo et al. [72]. Solvent control (MeOH) for MC-LR and a negative control (non-treated cells) were also included. All the dilutions were prepared in medium without serum. 

Cells were treated with the exposure solutions for 4, 24 and 48 h at 37 °C. The cytotoxicity biomarkers assayed were the total protein content (TP), evaluated following the procedure described by Bradford [73] and Pichardo et al. [74] and the reduction of the tetrazolium salt MTS, following the method described by Baltrop et al. [75]. Both assays were conducted three times independently.

### 5.4. Real-Time Quantitative PCR (qRT-PCR) Analysis after CYN Exposure

The expression of selected genes was analyzed by quantitative real time PCR (qPCR). HEK293 cells were seeded at 4 × 10^5^ cells/mL and incubated for 24 h at 37 °C in 5% CO_2_ to attach. Afterwards, the cells were exposed to fresh medium containing CYN at 0.5 and 5 µg/mL and incubated for 4 and 24 h. In each experiment, a positive control (30 µM B(a)P) was included following the procedure of Hercog et al. [32].

After CYN exposure the cells were washed with 1× PBS and total mRNA was isolated using TRIzol reagent according to Maisanaba et al. [76]. The concentration and purity of isolated mRNA was determined using NanoDrop 2000 Spectrophotometer (Thermo Fisher Scientific, Wilmington, DE, USA). All solutions needed for RNA isolation were prepared in RNase-free water. Three independent experiments were performed. The RNA was transcribed to cDNA using 1 µg of total RNA and cDNA High-Capacity Archive Kit, according to the manufacturer’s protocol.

Each specific gene product was amplified by Real time PCR using the ABI Prism 7000 sequence detector (Applied Biosystems, Foster City, CA, USA) according to the following parameters: 50 °C for 2 min, 95 °C for 10 min, 95 °C for 15 s (40 cycles) and 60 °C for 1 min (40 cycles).

Gene expression was quantified using PrimePCR™ Probe Assay (Bio-Rad, Hercules, CA, USA), and the following Gene Expression Assays were used: *CYP1A1* (cytochromeP450, family 1, subfamily A, polypeptide 1), qHsaCEP0058439; *CYP1A2* (cytochrome P450, family 1, subfamily A, polypeptide 2), qHsaCIP0029751; *TP53* (tumor protein P53), qHsaCEP0052284; *CDKN1A* (cyclin-dependent kinase inhibitor 1A) qHsaCIP0029411; *SOD1A* (superoxide dismutase 1), qHsaCIP0026883. *GPX1* (glutathione peroxidase 1) qHsaCEP0039727; *CAT* (catalase), qHsaCEP0051176; *BAX* (*BCL2*-associated X protein), qHsaCEP0040666; *BCL2* (B-cell CLL/lymphoma 2) and qHsaCIP0040441. 

Amplification of *GAPDH* probe (Human Endogenous Controls, qHsaCEP0041396) was performed as an internal control as described by Hercog et al. [32]. Data were analyzed using the ΔΔCt algorithm giving relative expression (RE) according to solvent control. Differences > 1.5-fold or <0.7 were considered as up/down-regulation, respectively.

### 5.5. Proteomic Analysis

#### 5.5.1. Sample Preparation

Proteomic studies were conducted in cells exposed to CYN, MC-LR and their combinations. HEK293 cells were seeded at 5 × 10^5^ cells/mL and incubated for 24 h at 37 °C in 5% CO_2_ to attach. Later, the cells were exposed to CYN (0.5 and 1.0 µg/mL), MC-LR (1 µg/mL) and their combinations for 24 h at 37 °C. Methanol (MeOH) was used as the solvent control.

After exposure, cells were homogenized in Tris (0.1 M), SDS (2% *w*/*v*), protease inhibitors and DTT (0.1 M) and brought to pH 7.6. Subsequently, they were sonicated (three cycles of 4 s at 60 Hz) and incubated for 2 h at room temperature. Samples were denatured with heat (3 min, 95 °C) and centrifuged at 16,000× *g*, for 20 min at 25 °C. Later, the supernatants were collected, and the total protein concentration was measured at 280 nm. 

Proteins were digested in accordance with the filter-aided sample preparation (FASP) method reported by Wisniewski et al. [77] using NMWL of 30 kDa. Protein samples (30 µg protein) were alkylated with IAA (0.05 M) and digested with trypsin at an enzyme-to-protein ratio of 1:100 (*w*/*w*) and incubated for 16 h at 37 °C. Centrifugal filtration was used to recover protein digests, acidified with FA (10% *v*/*v*), desalted and concentrated by reversed phase extraction (C18 tips). Prior to liquid chromatography coupled with tandem mass spectrometry (LC-MS/MS), the peptides were recovered in 0.1% FA (*v*/*v*) to the concentration of 0.06 µg/µL.

#### 5.5.2. LC-MS/MS

The LC-MS/MS was performed in a nano-LC coupled to a hybrid Ion trap mass spectrometer (LTQ Orbitrap Velos Pro-ETD) as described by Campos et al. [78] and Dominguez-Pérez et al. [79]. Peptides were separated by reverse phase chromatography on a 20 mm × 100 µm C18 precolumn followed by a 100 mm × 75 µm C18 column (particle size 5 µm, NanoSeparations, Nieuwkoop, The Netherlands) in a linear gradient of acetonitrile (2% to 95% *v*/*v*) in FA (0.1% *v*/*v*), at a flow rate of 0.3 µL/min (total elution time 70 min). Full scans were conducted at 30,000 resolution at a range of 380–2000 *m*/*z*. The top 20 most intense ions were isolated and fragmented with collision-induced fragmentation (CID) using normalized collision energy of 30%, isolation width of 2.0, activation time of 10 ms and a Q-value of 0.25. In total, 35 independent LC-MS/MS runs were conducted corresponding to the analysis of 35 biological samples.

#### 5.5.3. Protein Identification

Proteins were identified searching LTQ raw data against human protein sequences available at the Uniprot database (180503 sequences from UNIPROT downloaded June 2018) utilizing SEQUEST algorithm (Proteome Discoverer software, version 1.4, Thermo Scientific, Waltham, MA, USA) MS and MS/MS mass tolerances were set to 10 ppm and 0.6 Da, respectively. Trypsin was chosen for protein cleavage for one missed cleavage. Carbamidomethylation of cysteins were selected as static and methionine oxidation as dynamic modifications. 

Further evaluation and protein quantification was performed using the X!Tandem algorithm in Scaffold (version Scaffold 4.3.4, Proteome Software, Portland, OR, USA) [78]. Peptides were allowed if established at greater than 95.0% probability by the Scaffold local false discovery rate (FDR) algorithm, and proteins were accepted if established at greater than 99.9% probability. Proteins sharing significant peptide evidence were grouped into clusters grouped to satisfy the principles of parsimony. Protein label-free quantification was based on spectral counts information (normalized spectral abundance factors, NSAFs). Functional analysis of the differential proteins was performed using the web resource STRING [80]. 

STRING analysis was performed by selecting the Homo sapiens database for searching protein interaction evidence and to identify functional groups and pathways. Evidence accepted was from textmining, experiments, databases, co-expression, neighborhood, gene fusion and co-occurrence. Medium confidence (0.400) and max number of interactors (first shell)—no more than five defined the robustness of the analysis.

### 5.6. Statistical Analysis

Statistical analysis for the data of the cytotoxicity assays were conducted using analysis of variance (ANOVA), followed by Dunnett’s multiple comparison tests. The results were considered significant when * *p* < 0.05 and ** *p* < 0.01. For qRT-PCR analysis, statistical significance between treated groups and control group was determined by one-way analysis of variance (ANOVA) followed by Dunnett’s Multiple Comparison Test and the non-parametric Kruskal–Wallis test followed by Dunn’s multiple comparison test. 

Differences were considered to be significant from * *p* < 0.05, ** *p* < 0.01 and *** *p* < 0.001 with respect to the control group. Proteomics results were analyzed using one-way analysis of variance (ANOVA) followed by the Tukey–Kramer Multiple Comparisons Test. Statistical significance was considered at *p* < 0.01. All the analyses were performed using Multi Array Viewer version 4_9_0 (mev.tm4.org) and GraphPad InStat software (GraphPad Software Inc., La Jolla, CA, USA).

## Figures and Tables

**Table 1:** Effects of CYN on the expression of mRNA of selected genes involved in xenobiotic metabolism, DNA damage responsive, oxidative stress response and apoptosis/survival in HEK293 cells. The cells were exposed to CYN (0.5 and 5 µg/mL) for 4 and 24 h. B(a)P 30 µM was used as positive control.

Mechanisms Involved	Gene Symbol	CYN (µg/mL)	4 h	24 h	Entrez Gene Name
			Mean ± SD	Mean ± SD	
Xenobiotic Metabolism	*CYP1A1*	C-	1.13 ± 0.67	1.03 ± 0.33	Cytochrome P450 family 1 Subfamily A member 1
		0.5	1.11 ± 0.11	1.06 ± 0.35	
		5	1.99 ± 0.05 **	24.21 ± 11.92 ***	
		B(a)P 30 µM	1.03 ± 0.05	2.16 ± 0.33	
	*CYP1A2*	C-	1.01 ± 0.19	1.02 ± 0.25	Cytochrome P450 family 1 Subfamily A member 2
		0.5	0.90 ± 0.50	**0.68 ± 0.16**	
		5	0.87 ± 0.06	11.33 ± 0.17 *	
		B(a)P 30 µM	1.23 ± 0.40	1.23 ± 0.18	
DNA damage responsive	*TP53*	C-	1.05 ± 0.19	1.00 ± 0.12	Tumor protein P53
		0.5	0.86 ± 0.21	1.14 ± 0.25	
		5	0.98 ± 0.23	3.38 ± 0.31 **	
		B(a)P 30 µM	0.9 ± 0.20	1.22 ± 0.21	
	*CDKN1A*	C-	1.03± 0.30	1.01 ± 0.17	Cyclin Dependent Kinase Inhibitor 1A
		0.5	0.72 ± 0.14	**1.73 ± 0.40**	
		5	1.01 ± 0.56	10.57 ± 1.82 *	
		B(a)P 30 µM	0.88 ± 0.29	1.06 ± 0.68	
Oxidative stress	*SOD1*	C-	1.08 ± 0.47	1.01 ± 0.20	Superoxide dismutase 1
		0.5	1.08 ± 0.28	1.30 ± 0.25	
		5	2.04 ± 1.44 *	1.10 ± 0.68	
		B(a)P 30 µM	1.04 ± 0.13	1.05 ± 0.21	
	*CAT*	C-	1.08 ± 0.48	1.00 ± 0.13	Catalase
		0.5	1.41 ± 0.52	**1.45 ± 0.26**	
		5	**0.67 ± 0.83**	2.96 ± 1.35 ***	
		B(a)P 30 µM	1.21 ± 0.11	1.17 ± 0.10	
	*GPX1*	C-	1.05 ± 0.43	1.05 ± 0.39	Glutathione peroxidase 1
		0.5	1.04 ± 0.22	**2.07 ± 0.46**	
		5	0.89 ± 0.27	7.94 ± 2.35 ***	
		B(a)P 30 µM	1.22 ± 0.18	1.36 ± 0.58	
Apoptosis/survival	*BAX*	C-	1.02 ± 0.22	1.01 ± 0.14	Apoptosis regulator *BAX*.
		0.5	0.74 ± 0.17	1.30 ± 0.25	
		5	0.99 ± 0.30	0.92 ± 0.22	
		B(a)P 30 µM	0.87 ± 0.10	1.14 ± 0.24	
	*BCL2*	C-	1.01 ± 0.15	1.01 ± 0.16	B-cell CLL/lymphoma 2
		0.5	0.78 ± 0.21	1.33 ± 0.26 *	
		5	1.07 ± 0.02	3.85 ± 0.19 **	
		B(a)P 30 µM	0.79 ± 0.01	0.70 ± 0.13 *	

The results are expressed as relative mRNA expression normalized to the control group. Data are mean ± SD of three independent experiments. Significant differences between CYN-treated cells and the control group are indicated by * *p* < 0.05, ** *p* < 0.01 and *** *p* < 0.001 (One-way analysis of variance (ANOVA) with Dunnett’s Multiple Comparison Test and the non-parametric Kruskal–Wallis test followed by Dunn’s multiple comparison test). Bold values show the up- or down-regulation of genes.

**Table 2:** Differential protein expression in HEK293 cells exposed to CYN (0.5 and 1 µg/mL), MC-LR (1 µg/mL) or their combination of both toxins compared to the control. Quantitative expression is reported as normalized arbitrary units (AU). Significant differences between treated cells and the control group are indicated by * *p* < 0.05 and ** *p* < 0.01 (ANOVA).

AU														
Functional Category	Protein Name	Gene	C		0.5 CYN		1 CYN		1 MC-LR		0.5CYN + 1MCLR		1CYN + 1MC-LR	
			Mean	SD	Mean	SD	Mean	SD	Mean	SD	Mean	SD	Mean	SD
Cellular metabolism	Carbonyl reductase [NADPH] 1	*CBR1*	2.3 × 10^−4^	2.6 × 10^−4^	4.7 × 10^−4^	3.7 × 10^−4^	9.0 × 10^−4^	5.3 × 10^−4^	9.0 × 10^−4^	3.0 × 10^−4^	8.7 × 10^−4^	1.7 × 10^−4^	1.6 × 10^−3^ **	4.5 × 10^−4^
	Phosphoglucomutase-2	*PGM2*	1.0 × 10^−8^	0	1.0 × 10^−8^	9.0 × 10^−5^	1.0 × 10^−8^	0	1.0 × 10^−8^	0	2.8 × 10^−4^ **	1.7 × 10^−4^	1.0 × 10^−8^	0
Lipid metabolism	Prosaposin	*PSAP*	5.3 × 10^−4^	1.4 × 10^−4^	4.3 × 10^−4^	2.6 × 10^−4^	1.0 × 10^−8^ **	1.0 × 10^−4^	2.4 × 10^−4^	8.7 × 10^−5^	3.1 × 10^−4^	1.4 × 10^−4^	2.4 × 10^−4^ *	1.4 × 10^−4^
	3-ketoacyl-CoA thiolase, mitochondrial	*ACAA2*	6.4 × 10^−4^	2.7 × 10^−4^	5.7 × 10^−4^	3.9 × 10^−4^	1.0 × 10^−8^ *	1.3 × 10^−4^	4.3 × 10^−4^	2.7 × 10^−4^	1.4 × 10^−4^	2.7 × 10^−4^	1.0 × 10^−8^ *	0
Protein	Moesin	*MSN*	9.9 × 10^−4^	2.9 × 10^−4^	7.8 × 10^−4^	2.8 × 10^−4^	4.3 × 10^−4^ *	2.6 × 10^−4^	5.9 × 10^−4^	8.0 × 10^−5^	4.1 × 10^−4^ *	1.5 × 10^−4^	1.5 × 10^−4^ **	2.1 × 10^−4^
Cell adhesion	Integrin beta-1	*ITGB1*	2.5 × 10^−4^	8.1 × 10^−5^	1.0 × 10^−8^ *	7.9 × 10^−5^	1.0 × 10^−8^ *	6.6 × 10^−5^	1.0 × 10^−8^ *	7.4 × 10^−5^	1.0 × 10^−8^ **	0	1.0 × 10^−8^ **	0
	Fermitin family homolog 2	*FERMT2*	2.9 × 10^−4^	1.0 × 10^−4^	3.3 × 10^−4^	1.7 × 10^−4^	1.0 × 10^−8^	7.7 × 10^−5^	8.6 × 10^−5^	1.0 × 10^−4^	1.7 × 10^−4^	1.3 × 10^−4^	1.0 × 10^−8^ *	0
Protein Metabolism	Bleomycin hydrolase	*BLMH*	1.0 × 10^−8^	0	1.0 × 10^−8^	0	1.0 × 10^−8^	0	1.0 × 10^−8^	2.4 × 10^−4^	4.8 × 10^−4^ *	2.4 × 10^−4^	1.0 × 10^−8^	0
Protein regulation	E3 SUMO-protein ligase RanBP2	*RANBP2*	4.1 × 10^−5^	3.2 × 10^−5^	3.7 × 10^−5^	2.1 × 10^−5^	1.2 × 10^−4^ *	6.8 × 10^−5^	8.8 × 10^−5^	3.7 × 10^−5^	6.9 × 10^−5^	4.5 × 10^−5^	1.4 × 10^−4^	3.8 × 10^−4^
	E3 ubiquitin-protein ligase CHIP	*STUB1*	1.0 × 10^−8^	0	1.0 × 10^−8^	0	1.7 × 10^−4^	2.5 × 10^−4^	1.0 × 10^−8^	1.8 × 10^−4^	4.0 × 10^−4^ **	1.7 × 10^−4^	4.1 × 10^−4^	2.4 × 10^−4^
	Protein SGT1 homolog	*SUGT1*	4.3 × 10^−4^	1.8 × 10^−4^	3.9 × 10^−4^	3.1 × 10^−4^	1.0 × 10^−8^ *	0	1.0 × 10^−8^	1.8 × 10^−4^	3.6 × 10^−4^	2.7 × 10^−4^	1.0 × 10^−8^ *	0
	ATP-dependent Clp protease proteolytic subunit, mitochondrial	*CLPP*	1.0 × 10^−8^	0	1.0 × 10^−8^	0	1.0 × 10^−8^	0	4.3 × 10^−4^ **	3.6 × 10^−4^	1.0 × 10^−8^	0	1.0 × 10^−8^	0
Protein synthesis	Translation initiation factor eIF-2B subunit alpha	*EIF2B1*	1.0 × 10^−8^	0	1.0 × 10^−8^	0	1.0 × 10^−8^	0	1.0 × 10^−8^	0	3.6 × 10^−4^ *	1.8 × 10^−4^	1.0 × 10^−8^	2.0 × 10^−4^
	Treacle protein	*TCOF1*	4.0 × 10^−5^	8.0 × 10^−5^	1.0 × 10^−4^	1.0 × 10^−4^	1.0 × 10^−4^	5.0 × 10^−5^	2.0 × 10^−4^	1.0 × 10^−4^	3.0 × 10^−4^	6.2 × 10^−5^	3.0 × 10^−4^ *	9.3 × 10^−5^
	40S ribosomal protein S5	*RPS5*	7.0 × 10^−4^	3.0 × 10^−4^	7.0 × 10^−4^	3.0 × 10^−4^	1.0 × 10^−8^	3.0 × 10^−4^	1.0 × 10^−8^	3.0 × 10^−4^	5.0 × 10^−4^	3.0 × 10^−4^	1.0 × 10^−8^ *	0
	Cisplatin resistance-associated overexpressed protein, isoform CRA_b	*LUC7L3*	1.0 × 10^−8^	1.3 × 10^−4^	1.5 × 10^−4^	2.5 × 10^−4^	4.1 × 10^−4^ **	1.2 × 10^−4^	2.5 × 10^−4^	2.0 × 10^−5^	2.5 × 10^−4^	1.0 × 10^−4^	3.0 × 10^−4^	5.2 × 10^−5^
Protein transport	Coatomer subunit gamma-1	*COPG1*	7.0 × 10^−4^	5.1 × 10^−5^	3.2 × 10^−4^ *	1.9 × 10^−4^	1.8 × 10^−4^ *	2.5 × 10^−4^	2.2 × 10^−4^ *	1.8 × 10^−4^	6.3 × 10^−4^	1.6 × 10^−4^	1.7 × 10^−4^ **	1.8 × 10^−4^

## Data Availability

Not applicable.

## Supplementary Materials

The following supporting information can be downloaded at: https://www.mdpi.com/article/10.3390/toxins14070429/s1, Figure S1: Predicted functional associations of differentially expressed proteins in HEK293 cells.

## References

[1] Huisman J., Codd G.A., Paerl H.W., Ibelings B.W., Verspagen J.M.H., Visser P.M. (2018). Cyanobacterial blooms. Nat. Rev. Microbiol..

[2] Funari E., Testai E. (2008). Human health risk assessment related to cyanotoxins exposure. Crit. Rev. Toxicol..

[3] Catherine A., Bernard C., Spoof L., Bruno M., Meriluoto J., Spoof L., Cood G.A. (2017). Mycrocistins and nodularins. Handbook of Cyanobacterial Monitoring and Cyanotoxin Analysis.

[4] Bouaïcha N., Miles C.O., Beach D.G., Labidi Z., Djabri A., Benayache N.Y., Nguyen-Quang T. (2019). Structural diversity, characterization and toxicology of microcystins. Toxins.

[5] Diez-Quijada L., Puerto M., Gutiérrez-Praena D., Llana-Ruiz-Cabello M., Jos Á., Cameán A.M. (2019). Microcystin-RR: Occurrence, content in water and food and toxicological studies. A review. Environ. Res..

[6] Diez-Quijada L., Prieto A.I., Gumán-Guillé R., Jos Á., Cameán A.M. (2019). Occurrence and toxicity of microcystin congeners other than MC-LR and MC-RR: A review. Food Chem. Toxicol..

[7] Buratti F.M., Manganelli M., Vichi S., Stefanelli M., Scardala S., Testai M., Funari E. (2017). Cyanotoxins: Producing organisms, occurrence, toxicity, mechanism of action and human health toxicological risk evaluation. Arch. Toxicol..

[8] Mackintosh C., Beattie K.A., Klumpp S., Cohen P., Codd G.A. (1990). Cyanobacterial microcystin-LR is a potent and specific inhibitor of protein phosphatases 1 and 2 A from both mammals and higher plants. FEBS Lett..

[9] Ingested Nitrate and Nitrite, and Cyanobacterial Peptide Toxins. https://www.ncbi.nlm.nih.gov/books/NBK326544/pdf/Bookshelf_NBK326544.pdf.

[10] Ohtani I., Moore R.E., Runnegar M.T.C. (1992). Cylindrospermopsin—A potent hepatotoxin from the blue green alga *Cylindrospermopsis raciborskii*. J. Am. Chem. Soc..

[11] Oliveira F., Diez-Quijada L., Turkina M.V., Morais J., Felpeto A.B., Azevedo J., Jos A., Camean A.M., Vasconcelos V., Martins J.C. (2020). Physiological and metabolic responses of marine mussels exposed to toxic cyanobacteria *Microcystis aeruginosa* and *Chrysosporum ovalisporum*. Toxins.

[12] Terao K., Ohmori S., Igarashi K., Ohtani I., Watanabe M.F., Harada K.I., Ito E., Watanabe M. (1994). Electron microscopic studies on experimental poisoning in mice induced by cylindrospermopsin isolated from blue-green alga *Umezakia natans*. Toxicon.

[13] Runnegar M.T., Kong S.-M., Zhong Y.-Z., Lu S.C. (1995). Inhibition of reduced glutathione synthesis by cyanobacterial alkaloid cylindrospermopsin in cultured rat hepatocytes. Biochem. Pharmacol..

[14] Froscio S.M., Humpage A.R., Burcham P.C., Falconer I.R. (2003). Cylindrospermopsin-induced protein synthesis inhibition and its dissociation from acute toxicity in mouse hepatocytes. Environ. Toxicol. Int. J..

[15] Hawkins P.R., Runnegar M.T., Jackson A.R., Falconer I.R. (1985). Severe hepatotoxicity caused by the tropical cyanobacterium (blue-green alga) *Cylindrospermopsis raciborskii* (Woloszynska) Seenaya and Subba Raju isolated from a domestic water supply reservoir. Appl. Environ. Microbiol..

[16] Žegura B. (2016). An overview of the mechanisms of microcystin-LR genotoxicity and potential carcinogenicity. Mini Rev. Med. Chem..

[17] Hinojosa M.G., Gutiérrez-Praena D., Prieto A.I., Guzmán-Guillén R., Jos Á., Cameán A.M. (2019). Neurotoxicity induced by microcystins and cylindrospermopsin: A review. Sci. Total Environ..

[18] Xu S., Yi X., Liu W., Zhang C., Massey I.Y., Yang F., Tian L. (2020). A review of nephrotoxicity of microcystins. Toxins.

[19] Piyathilaka M.A.P.C., Pathmalal M.M., Tennekoon K.H., de Silva B.G.D.N.K., Samarakoon S.R., Chanthirika S. (2015). Microcystin-LR-induced cytotoxicity and apoptosis in human embryonic kidney and human kidney adenocarcinoma cell lines. Microbiology.

[20] Prieto A.I., Pichardo S., Jos Á., Moreno I., Cameán A.M. (2007). Time-dependent oxidative stress responses after acute exposure to toxic cyanobacterial cells containing microcystins in Tilapia fish (*Oreochromis niloticus*) under laboratory conditions. Aquat. Toxicol..

[21] Atencio L., Moreno I., Prieto A.I., Moyano R., Molina A.M., Cameán A.M. (2008). Acute effects of microcystins MC-LR and MC-RR on acid and alkaline phosphatase activities and pathological changes in intraperitoneally exposed Tilapia fish (*Oreochromis* sp.). Toxicol. Pathol..

[22] Froscio S.M., Cannon E., Lau H.M., Humpage A.R. (2009). Limited uptake of the cyanobacterial toxin cylindrospermopsin by Vero cells. Toxicon.

[23] Puerto M., Jos Á., Pichardo S., Gutiérrez-Praena D., Cameán A.M. (2011). Acute effects of pure cylindrospermopsin on the activity and transcription of antioxidant enzymes in tilapia (*Oreochromis niloticus*) exposed by gavage. Ecotoxicology.

[24] Gutiérrez-Praena D., Jos Á., Pichardo S., Moyano R., Blanco A., Monterde J.G., Cameán A.M. (2012). Time-dependent histopathological changes induced in Tilapia (*Oreochromis niloticus*) after acute exposure to pure cylindrospermopsin by oral and intraperitoneal route. Ecotoxicol. Environ. Saf..

[25] Guzmán-Guillén R., Prieto A.I., Vasconcelos V.M., Cameán A.M. (2013). Cyanobacterium producing cylindrospermopsin cause oxidative stress at environmentally relevant concentrations in sub-chronically exposed tilapia (*Oreochromis niloticus*). Chemosphere.

[26] Testai E., Buratti F.M., Funari E., Manganelli M., Vichi S., Arnich N., Biré R., Fessard V., Sialehaamoa A.A. (2016). Review and analysis of occurrence, exposure and toxicity of cyanobacteria toxins in food. EFSA Support. Publ..

[27] Minasyan A., Christophoridis C., Wilson A.E., Zervou S.-K., Kaloudis T., Hiskia A. (2018). Diversity of cyanobacteria and the presence of cyanotoxins in the epilimnion of Lake Yerevan (Armenia). Toxicon.

[28] León C., Boix C., Beltrán E., Peñuela G., López F., Sancho J.V., Hernández F. (2019). Study of cyanotoxin degradation and evaluation of their transformation products in surface waters by LC-QTOF MS. Chemosphere.

[29] Li H., Gu X., Chen H., Mao Z., Shen R., Zeng Q., Ge Y. (2022). Co-occurrence of multiple cyanotoxins and taste-and-odor compounds in the large eutrophic Lake Taihu, China: Dynamics, driving factors, and challenges for risk assessment. Environ. Pollut..

[30] Gutiérrez-Praena D., Guzmán-Guillén R., Pichardo S., Moreno F.J., Vasconcelos V., Jos Á., Cameán A.M. (2019). Cytotoxic and morphological effects of microcystin-LR, cylindrospermopsin, and their combinations on the human hepatic cell line HepG2. Environ. Toxicol..

[31] Hinojosa M.G., Prieto A.I., Gutiérrez-Praena D., Moreno F.J., Cameán A.M., Jos Á. (2019). Neurotoxic assessment of microcystin-LR, cylindrospermopsin and their combination on the human neuroblastoma SH-SY5Y cell line. Chemosphere.

[32] Hercog K., Maisanaba S., Filipic M., Jos Á., Cameán A.M., Žegura B. (2017). Genotoxic potential of the binary mixture of cyanotoxins microcystin-LR and cylindrospermopsin. Chemosphere.

[33] Díez-Quijada L., Prieto A.I., Puerto M., Jos Á., Cameán A.M. (2019). In vitro mutagenic and genotoxic assessment of a mixture of the cyanotoxins microcystin-LR and cylindrospermopsin. Toxins.

[34] Humpage A.R., Falconer I.R. (2003). Oral toxicity of the cyanobacterial toxin cylindrospermopsin in male Swiss Albino mice: Determination of no observed adverse effect level for deriving a drinking water guideline value. Environ. Toxicol..

[35] Fischer A., Hoeger S.J., Stemmer K., Feurstein D.J., Knobeloch D., Nussler A., Dietrich D.R. (2010). The role of organic anion transporting polypeptides (OATPs/SLCOs) in the toxicity of different microcystin congeners in vitro: A comparison of primary human hepatocytes and OATP-transfected HEK293 cells. Toxicol. Appl. Pharmacol..

[36] Fan H., Cai Y., Xie P., Xiao W., Che J., Ji W., Zhao S. (2014). Microcystin-LR stabilizes c-myc protein by inhibiting proteinphosphatase 2A in HEK293 cells. Toxicology.

[37] Takumi S., Shimono T., Ikema S., Hotta Y., Chigwechokha P.K., Sugiyama Y., Hashimoto M., Furukawa T., Komatsu M. (2017). Overexpression of carboxylesterase contributes to the attenuation of cyanotoxin microcystin-LR toxicity. Comp. Biochem. Physiol. C.

[38] Ray S.D., Farris F.F., Hartman A.C., Wexler P. (2014). Hormesis. Encyclopedia of Toxicology.

[39] Li T., Huang P., Liang J., Fu W., Guo Z., Xu L. (2011). Microcystin-LR (MCLR) induces a compensation of PP2A activity mediated by α4 protein in HEK293 cells. Int. J. Biol. Sci..

[40] Dias E., Andrade M., Alverca E., Pereira P., Batoréu M.C.C., Jordan P., Silva M.J. (2009). Comparative study of the cytotoxic effect of microcistin-LR and purified extracts from *Microcystis aeruginosa* on a kidney cell line. Toxicon.

[41] Froscio S.M., Fanok S., Humpage A.R. (2009). Cytotoxicity screening for the cyanobacterial toxin cylindrospermopsin. J. Toxicol. Environ. Health A.

[42] Moraes A.C.N., Freire D.S., Habibi H., Lowe J., Magalhães V.F. (2021). Cylindrospermopsin impairs tubular transport function in kidney cells LLC-PK1. Toxicol Lett..

[43] Žegura B., Gajski G., Štraser A., Garaj-Vrhovac V. (2011). Cylindrospermopsin induced DNA damage and alteration in the expression of genes involved in the response to DNA damage, apoptosis and oxidative stress. Toxicon.

[44] Štraser A., Filipič M., Žegura B. (2011). Genotoxic effects of the cyanobacterial hepatotoxin cylindrospermopsin in the HepG2 cell line. Arch. Toxicol..

[45] Štraser A., Filipič M., Žegura B. (2013). Cylindrospermopsin induced transcriptional responses in human hepatoma HepG2 cells. Toxicol. Vitro.

[46] Hercog K., Štampar M., Štern A., Filipič M., Žegura B. (2020). Application of advanced HepG2 3D cell model for studying genotoxic activity of cyanobacterial toxin cylindrospermopsin. Environ. Pollut..

[47] Zhang Q., Wang L., Chen G., Wang M., Hu T. (2021). Cylindrospermopsin impairs vascular smooth muscle cells by P53-mediated apoptosis due to ROS overproduction. Toxicol. Lett..

[48] Wang L., Chen G., Xiao G., Han L., Wang Q., Hu T. (2020). Cylindrospermopsin induces abnormal vascular development through impairing cytoskeleton and promoting vascular endothelial cell apoptosis by the Rho/ROCK signaling pathway. Environ. Res..

[49] Díez-Quijada L., Hercog K., Štampar M., Filipič M., Cameán A.M., Jos Á., Žegura B. (2020). Genotoxic effects of cylindrospermopsin, microcystin-LR and their binary mixture in human hepatocellular carcinoma (HepG2) cell line. Toxins.

[50] Androutsopoulos V.P., Tsatsakis A.M., Spandidos D.A. (2009). Cytochrome P450 *CYP1A1*: Wider roles in cancer progression and prevention. BMC Cancer.

[51] Lee W.Y.W., Zhou X., Or P.M.Y., Kwan Y.W., Yeung J.H.K. (2012). Tanshinone I increases *CYP1A2* protein expression and enzyme activity in primary rat hepatocytes. Phytomedicine.

[52] Vogelstein B., Lane D., Levine A.J. (2000). Surfing the p53 network. Nature.

[53] Bain P., Shaw G., Patel B. (2007). Induction of p53-regulated gene expression in human cell lines exposed to the cyanobacterial toxin cylindrospermopsin. J. Toxicol. Environ. Health A.

[54] Michael D., Oren M. (2002). The p53 and Mdm2 families in cancer. Curr. Opin. Genet. Dev..

[55] Pichardo S., Cameán A.M., Jos A. (2017). In vitro toxicological assessment of cylindrospermopsin: A review. Toxins.

[56] Fridovich I. (1978). The biology of oxygen radicals. Science.

[57] Ighodaro O.M., Akinloye O.A. (2018). First line defence antioxidants superoxide dismutase (SOD), catalase (*CAT*) and glutathione peroxidase (GPX): Their fundamental role in the entire antioxidant defence grid. Alex. J. Med..

[58] Galasso M., Gambino S., Romanelli M.G., Donadelli M., Scupoli M.T. (2021). Browsing the oldest antioxidant enzyme: Catalase and its multiple regulation in cancer. Free Radic. Biol. Med..

[59] Araujo Eleutherio E.C., Silva Magalhaes R.S., de Araújo Brasil A., Monteiro Neto J.R., de Holanda Paranhos L. (2021). *SOD1*, more than just an antioxidant. Arch. Biochem. Biophys..

[60] Stolwijk J.M., Falls-Hubert K.C., Searby C.C., Wagner B.A., Buettner G.R. (2020). Simultaneous detection of the enzyme activities of *GPx1* and *GPx4* guide optimization of selenium in cell biological experiments. Redox Biol..

[61] Araújo M.J., Sousa M.L., Felpeto A.B., Turkina M.V., Fonseca E., Martins J.C., Vasconcelos V., Campos A. (2021). Comparison of sample preparation methods for shotgun proteomic studies in aquaculture species. Proteomes.

[62] Campos A., Tedesco S., Vasconcelos V., Cristobal S. (2012). Proteomic research in bivalves: Towards the identification of molecular markers of aquatic pollution. J. Proteom..

[63] UniProt. https://www.uniprot.org/uniprot/P16152.

[64] Puerto M., Campos A., Prieto A., Cameán A., de Almeida A.M., Coelho A.V., Vasconcelos V. (2011). Differential protein expression in two bivalve species; *Mytilus galloprovincialis* and *Corbicula fluminea*; exposed to *Cylindrospermopsis raciborskii* cells. Aquat. Toxicol..

[65] Menezes C., Valerio E., Días E., Gowder S. (2013). The kidney vero-E6 cell line: A suitable model to study the toxicity of microcystins. New Insights into Toxicity and Drug Testing.

[66] Fu W., Yu Y., Xu L. (2009). Identification of temporal differentially expressed protein responses to microcystin in human amniotic epithelial cells. Chem. Res. Toxicol..

[67] Zhao S., Xie P., Chen J., Liu L., Fan H. (2016). A proteomic study on liver impairment in rat pups induced by maternal microcystin-LR exposure. Environ. Pollut..

[68] Li G., Cai F., Yan W., Li C., Wang J. (2012). A proteomic analysis of MCLR-induced neurotoxicity: Implications for Alzheimer’s disease. Toxicol. Sci..

[69] Li G., Yan W., Qiao Q., Chen J., Cai F., He Y., Zhang X. (2012). Global effects of subchronic treatment of microcystin-LR on rat splenetic protein levels. J. Proteom..

[70] Chen T., Wang Q., Cui J., Yang W., Shi Q., Hua Z., Ji J., Shen P. (2005). Induction of apoptosis in mouse liver by microcystin-LR: A combined transcriptomic, proteomic, and simulation strategy. Mol. Cell. Proteom..

[71] Liebel S., Regina Grötzner S., Dietrich Moura Costa D., Antônio Ferreira Randi M., Alberto de Oliveira Ribeiro C., Filipak Neto F. (2016). Cylindrospermopsin effects on protein profile of HepG2 cells. Toxicol. Mech. Methods.

[72] Pichardo S., Jos Á., Zurita J.L., Salguero M., Cameán A.M., Repetto G. (2005). The use of the fish cell lines RTG-2 and PLHC-1 to compare the toxic effects produced by microcystins LR and RR. Toxicol. Vitro.

[73] Bradford M.M. (1976). A rapid and sensitive method for the quantitation of microgram quantities of protein utilizing the principle of protein-dye binding. Anal. Biochem..

[74] Pichardo S., Jos A., Zurita J.L., Salguero M., Cameán A.M., Repetto G. (2007). Acute and subacute toxic effects produced by microcystin-YR on the fish cell lines RTG-2 and PLHC-1. Toxicol. Vitro.

[75] Baltrop J.A., Owen T.C., Cory A.H., Cory J.G. (1991). 5-((3-carboxyphenyl)-3-(4,5-dimethylthiazolyl)-3-(4-sulfophenyl)) tetrazolium, inner salt (MTS) and related analogs of 2-(4,5-dimethylthiazolyl)-2,5-diphenylterazolium bromide (MTT) reducing to purple water soluble formazan as cell-viability indicators. Bioorg. Med. Chem. Lett..

[76] Maisanaba S., Hercog K., Filipic M., Jos A., Zegura B. (2016). Genotoxic potential of montmorillonite clay mineral and alteration in the expression of genes involved in toxicity mechanisms in the human hepatoma cell line HepG2. J. Hazard. Mater..

[77] Wiśniewski J.R., Zougman A., Nagaraj N., Mann M. (2009). Universal sample preparation method for proteome analysis. Nat. Methods.

[78] Campos A., Danielsson G., Farinha A.P., Kuruvilla J., Warholm P., Cristobal S. (2016). Shotgun proteomics to unravel marine mussel (*Mytilus edulis*) response to long-term exposure to low salinity and propranolol in a Baltic Sea microcosm. J. Proteom..

[79] Domínguez-Pérez D., Rodríguez A.A., Osorio H., Azevedo J., Castañeda O., Vasconcelos V., Antunes A. (2017). Microcystin-LR detected in a low molecular weight fraction from a crude extract of *Zoanthus sociatus*. Toxins.

[80] String. https://string-db.org.

