# Supplementary material for: In Vitro Toxicity Evaluation of Cyanotoxins Cylindrospermopsin and Microcystin-LR on Human Kidney HEK293 Cells
Source: Toxins (Basel). 2022 Jun 23;14(7):429. doi: 10.3390/toxins14070429 (PMC9316492; doi:10.3390/toxins14070429)
Supplement: Supplementary file 1 [file toxins-14-00429-s001.zip › toxins-1761058-supplementary.pdf]

toxins

In Vitro Toxicity Evaluation of Cyanotoxins Cylindrospermopsin and Microcystin-LR on Human Kidney HEK293 Cells

Leticia Diez-Quijada, María Puerto, Daniel Gutiérrez-Praena, Maria V. Turkina, Alexandre Campos, Vitor Vasconcelos, Ana M. Cameán and Ángeles Jos

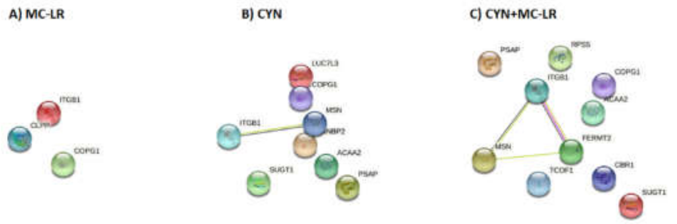

Figure S1. Predicted functional associations of differentially expressed proteins in HEK293 cells.
